# Automatic per-segment analysis of myocardial perfusion MRI

**DOI:** 10.1186/1532-429X-13-S1-P34

**Published:** 2011-02-02

**Authors:** Marie-Pierre Jolly, Hui Xue, Xiaoguang Lu, Christoph Guetter, Peter Kellman, Li-Yueh Hsu, Andrew E Arai, Sven Zuehlsdorff, Jens Guehring

**Affiliations:** 1 Siemens Corporate Research, Princeton, NJ, USA; 2 National Institutes of Health, Bethesda, MD, USA; 3 Siemens Medical Solutions, Chicago, IL, USA

## Background

We have previously proposed an automatic solution to calculate semi-quantitative measurbes of myocardial perfusion and to present them to the user as parametric maps. Such maps have high spatial resolution which can potentially be an advantage when evaluating small regions of myocardium. On the other hand, per-segment analyses potentially benefit from higher SNR and improved clarity of presentation.

## Objective

To demonstrate feasibility of automatic semi-quantitative per-segment analysis of myocardial perfusion time series according to the AHA 17-segment model.

## Methods

Five subjects with suspected CAD were scanned at rest and during stress on Siemens 1.5T MAGNETOM Espree/Avanto scanners using two established perfusion sequence techniques (TurboFLASH and GRE-EPI). All datasets were corrected for myocardial motion and surface coil inhomogeneity. Image noise was suppressed using adaptive filtering. Pixel-wise semi-quantitative maps were computed according to [1].

Starting from the most basal slice, the two RV insertion points and the LV center point were automatically determined by a landmark detection algorithm, based on probabilistic boosting trees and marginal space learning. It was trained with a database of 373 manually annotated time series.

The obtained center point is used to convert the image into polar space to extract the endocardial and epicardial boundaries. The contours are recovered by finding the shortest path using Dijkstra’s algorithm according to a gradient-based cost function. The anterior RV insertion point is used to orient the segment model.

Quantitative validation was performed on all 20 slices. LV and myocardium boundaries were manually delineated. Landmark detection was evaluated by measuring the distance between the detected RV insertion points and manual annotation. Myocardial segmentation was evaluated by comparing the automatic results against manual delineation.

## Results

**Figure 1:**
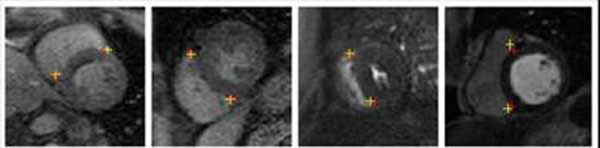
Examples of myocardial landmark detection. BLUE: manually picked RV insertion points, RED: automatically detected points. The joint spatio-temporal classifier is robust against different imaging sequences and geometry.

The overall effectiveness of the solution was confirmed in all five patients by visual assessment. The mean error for landmark detection was 4.5±2.6mm (Figure 1). This defines the position of the segment model accurately. The Dice ratio for myocardial segmentation was 0.93±0.025. Median MBE (minimal distance between segmentation and reference) was 1.13/1.02mm for endo/epi.

**Figure 2:**
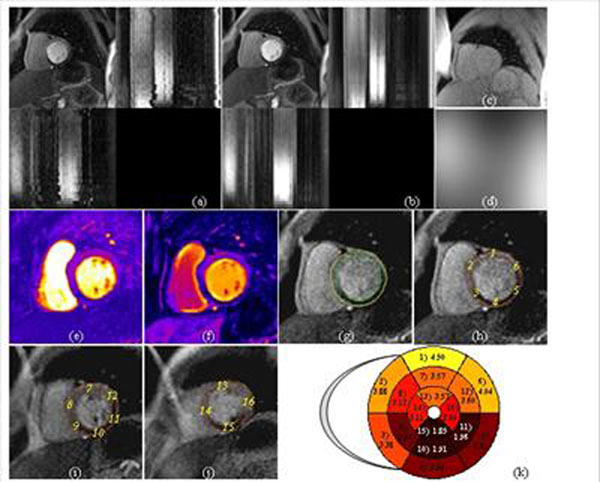
Automatic Per-Segment Analysis of Myocardial Perfusion MRI (with perfusion deficit). (a) Original time series with perfusion deficit; (b) Processed series after motion correction, B1 surface coil inhonogeneity correction and adaptive noise suppression; (c) PD images; (d) estimated inhomogeneity field; (e-f) Estimated parameters maps (e: up-slope; f: area-under-curve); (g) Segmentation result with detected RV insertion and LV center points; (h-j) Per-segment delineation for basal (h), medial (i), and apical (j) slices; (k) AHA 17-segment model with averaged up-slope values.

**Figure 3:**
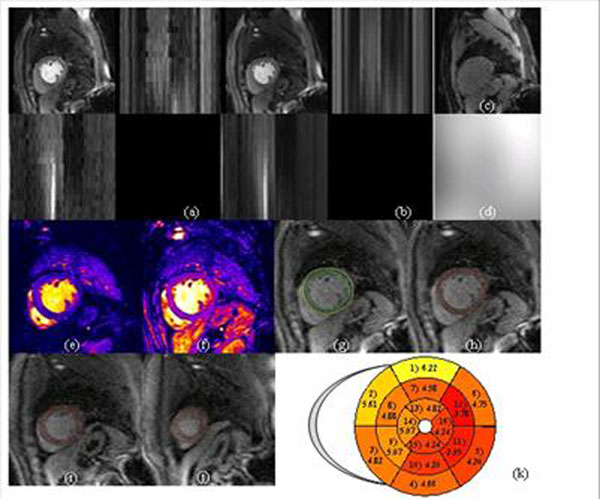
Automatic Per-Segment Analysis of Myocardial Perfusion MRI (without perfusion deficit). (a) Original time series without perfusion deficit; (b) Processed series after motion correction, B1 surface coil inhonogeneity correction and adaptive noise suppression; (c) PD images; (d) estimated inhomogeneity field; (e-f) Estimated parameters maps (e: up-slope; f: area-under-curve); (g) Segmentation result with detected RV insertion and LV center points; (h-j) Per-segment delineation for basal (h), medial (i), and apical (j) slices; (k) AHA 17-segment model with averaged up-slope values.

The typical performance of the proposed workflow is illustrated in Figure 2 and Figure 3. In both cases, the parametric maps and per-segment results correctly characterize the status of the myocardium.

## Conclusion

Feasibility of automatic semi-quantitative per-segment analysis of myocardial perfusion time series was successfully demonstrated with data from five subjects with images of rather good quality, warranting a larger clinical study of this method.
